# Aligning insurance payment with public value: outcome-based payment for traditional Chinese medicine advantage diseases under China’s DRG/DIP reform

**DOI:** 10.3389/fpubh.2026.1902781

**Published:** 2026-10-21

**Authors:** Zhongyang Pei, Anxia Hu, Zhiguang Duan

**Affiliations:** 1 School of Management, Shanxi Medical University, Taiyuan, Shanxi, China; 2 School of Health Services and Management, Shanxi University of Chinese Medicine, Taiyuan, Shanxi, China

**Keywords:** China, DRG/DIP reform, medical insurance, outcome-based payment, patient-centered public value, traditional Chinese medicine, value-based healthcare

## Abstract

**Background:**

China’s DRG/DIP payment reform has strengthened prospective case-based reimbursement, but conventional grouping metrics may not fully capture the patient-perceived and TCM-specific value of Traditional Chinese Medicine (TCM) for TCM-advantage diseases.

**Objective:**

This study evaluated whether outcome-based payment for TCM-advantage diseases improved patient-centered public value compared with conventional DRG/DIP payment.

**Methods:**

A multicenter, mixed-methods quasi-experimental study was conducted using medical insurance settlement data, discharge abstracts, electronic medical records, patient questionnaires, and semi-structured interviews. The administrative cohort included 7,985 inpatient cases across five TCM-advantage disease cohorts, and the patient-reported component included 545 questionnaires. Ten stakeholders were interviewed to contextualize the quantitative findings. A Patient-Centered Public Value Index (PCPVI) was constructed across five dimensions and analyzed using propensity score matching and difference-in-differences estimation.

**Results:**

Outcome-based payment was associated with a 4.85-point increase in the PCPVI (95% CI: 3.32 to 6.38; *p* < 0.001). It was also associated with reductions of 520 RMB in total hospitalization expenditure and 300 RMB in out-of-pocket payment per admission. The reimbursement ratio and TCM treatment cost proportion increased by 2.40 and 4.90 percentage points, respectively. The insurance fund payment estimate was −130 RMB (95% CI: −361 to 101; *p* = 0.270), and the EQ-5D-5L estimate was 0.021 (95% CI: −0.001 to 0.043; *p* = 0.061). Neither was statistically significant. No statistically significant differences were detected in 30-day readmission or in-hospital complications. Patient satisfaction and perceived insurance benefit were higher in the outcome-based payment group.

**Conclusion:**

In the Taiyuan study setting, outcome-based payment was associated with better financial protection, a higher PCPVI and more favorable patient experience. Increased TCM expenditure share indicates a change in service composition, rather than direct evidence of TCM-specific therapeutic efficacy. Effects on generic health-related quality of life, insurance fund payment and short-term safety outcomes remain uncertain. Longer follow-up and external validation are needed before drawing conclusions about fund sustainability or transferability.

## 1 Introduction

China has expanded diagnosis-related group (DRG) and diagnosis-intervention packet (DIP) payment as part of its medical insurance payment reform; the national grouping schemes were updated to version 2.0 in July 2024 (1). These case-based arrangements alter the incentives associated with fee-for-service reimbursement, although provider responses vary across settings (2, 3). Evaluations have reported differences in costs, length of stay and care quality across hospital levels and clinical specialties (4, 5). Their application to Traditional Chinese Medicine (TCM) therefore requires attention to both resource use and the services delivered.

In this study, TCM-advantage diseases refer to conditions included in the local TCM-oriented payment evaluation; this designation does not itself establish superior clinical efficacy. Research in TCM hospitals has identified changes in service composition and expenditure on TCM techniques after DRG reform (6, 7). Effects on length of stay and hospitalization costs have also differed between secondary and tertiary TCM hospitals (8). These findings motivate an evaluation that considers patient outcomes and experience alongside costs, while distinguishing expenditure on TCM from evidence of therapeutic benefit.

Outcome-based payment links reimbursement incentives to patient outcomes and care quality, extending evaluation beyond expenditure containment (9–11). Value-based healthcare emphasizes outcomes relative to costs, whereas public value also asks whether the purposes and distribution of publicly financed services are justified. Moore’s strategic triangle connects substantive public value, legitimacy and support, and operational capacity (12). Applied to medical insurance, these elements concern patient benefit, the authorization and accountability of payment rules, and the capacity of insurers and hospitals to implement them. This is a conceptual application of the framework, not an empirical demonstration that all three elements were achieved.

Patient-level gains contribute to public value when they advance collectively justified goals through the responsible use of pooled resources; individual satisfaction alone is insufficient (13). We therefore interpret financial protection and care experience as patient-level evidence relevant to public value, alongside questions of equitable access, accountability and opportunity cost. TCM provision has no automatic public-value status: its contribution depends on clinical appropriateness, patient benefit and fair allocation. The Patient-Centered Public Value Index (PCPVI) operationalizes a patient-centered subset of this broader concept. It does not directly measure population-wide equity, public legitimacy or long-term fiscal sustainability.

Chinese payment-reform evaluations have examined financial protection and service utilization among low-income patients (14), inpatient cost structure (15), and hospitalization expenses across payment groups and surgical specialties (16, 17). Other studies have assessed patient selection and provider responses (18, 19). Quality findings are heterogeneous: evaluations have reported changes that differ by hospital setting and outcome (20, 21), whereas a general-surgery study found no significant changes in the measured adverse clinical outcomes (22). Hospital performance studies also indicate that immediate changes and subsequent trends need to be distinguished (23). This literature supports evaluating cost, quality and patient experience together; it does not establish the effects of the specific local outcome-based payment arrangement examined here. The present study applies a multidimensional patient-centered framework to that setting.

To address this evaluation need, this study developed a patient-centered public value evaluation framework for outcome-based payment of TCM-advantage diseases under DRG/DIP reform. It integrated medical insurance settlement data, hospital discharge abstracts, electronic medical records, patient questionnaires, and stakeholder interviews. The quantitative component estimated policy effects on cost, quality, efficiency, TCM service use, and patient-reported outcomes. The qualitative component examined how patients, clinicians, and insurance managers perceived the implementation mechanisms and practical barriers of outcome-based payment.

The central hypothesis was that outcome-based payment for TCM-advantage diseases would improve patient-centered public value by promoting standardized TCM pathways, optimizing cost structure, reducing patient financial burden, and improving patient-perceived benefit without increasing avoidable readmission or compromising care quality. Specifically, this study aimed to: first, construct a Patient-Centered Public Value Index for TCM-advantage disease payment evaluation; second, compare outcome-based payment with conventional DRG/DIP payment using real-world claims and discharge data; third, evaluate patient-reported outcomes and satisfaction among 545 patients; and fourth, explain implementation mechanisms through semi-structured interviews with 10 key stakeholders.

## 2 Methods

### 2.1 Study design

This study adopted a multicenter, mixed-methods, quasi-experimental design to evaluate outcome-based payment for TCM-advantage diseases under China’s DRG/DIP payment reform. The quantitative component combined medical insurance settlement data, hospital discharge abstracts, electronic medical records, and patient-reported questionnaires. The qualitative component used semi-structured interviews to explain implementation mechanisms and contextual factors.

The study was designed to assess whether outcome-based payment generated patient-centered public value beyond conventional cost containment. The evaluation therefore focused on medical expenditure, insurance payment, quality of care, TCM-specific service value, and patient-reported benefit.

### 2.2 Policy setting and study period

The study was conducted in Taiyuan, Shanxi Province, China, across eight hospitals (four tertiary and four secondary hospitals) within its local TCM-advantage disease payment framework. The exposure was the designated outcome-based payment pathway for selected TCM-advantage diseases within the DRG/DIP environment. January 1, 2024 was used as the policy cut-off for the analysis. Taiyuan was treated as a local implementation setting; it was not assumed to represent other provinces in disease coverage, reimbursement rules or insurance fund capacity.

The pre-policy period was January 1, 2022 to December 31, 2023, and the post-policy period was January 1, 2024 to December 31, 2025. These periods provided 2 years of observations on either side of the analytical cut-off. Concurrent changes in payment rules remain a potential source of confounding: national DRG/DIP version 2.0 guidance was released during 2024 (1). Its local implementation timing and any differential exposure across payment groups should be considered when interpreting the estimates.

### 2.3 Disease cohorts

Five TCM-advantage disease cohorts were included. Eligible disease cohorts were required to meet four conditions: inclusion in the local TCM-advantage disease payment catalogue, clear correspondence between TCM disease coding and Western medical diagnosis coding, stable clinical pathway, and adequate inpatient case volume.

Each disease cohort contained patients exposed to the outcome-based TCM payment pathway and comparable patients managed under conventional DRG/DIP payment. For difference-in-differences (DID) estimation, the payment-group indicator identified the pathway or hospital-disease combination exposed to the outcome-based policy after the cut-off point, while disease cohort fixed effects controlled for baseline differences in disease severity, clinical pathway, cost structure, and expected recovery.

### 2.4 Data sources and linkage

The administrative dataset consisted of medical insurance settlement records and hospital discharge abstracts. Insurance settlement records provided payment type, DRG/DIP group, total hospitalization expenditure, medical insurance fund payment, out-of-pocket payment, reimbursement ratio, and itemized expenditure. Discharge abstracts provided age, sex, admission route, principal diagnosis, secondary diagnoses, procedures, length of stay, discharge status, and readmission records.

Electronic medical records were used to verify TCM diagnosis, TCM syndrome information, TCM treatment items, and disease-specific clinical indicators. Data linkage was performed using encrypted patient identifiers, hospitalization number, admission date, and discharge date. Western diagnoses were standardized using the medical-insurance version of International Classification of Diseases (ICD) codes, and TCM diagnoses were standardized using TCM disease codes (TCD). Coding standardizes diagnoses within each coding system, whereas mapping specifies the correspondence between ICD and TCD codes. A clear correspondence at the disease-cohort level was required for selection of the target cohorts. Record-level availability of valid ICD-TCD mapping was assessed separately as a data-completeness indicator.

### 2.5 Study population and sample size

A total of 7,985 inpatient cases were included in the medical insurance settlement and discharge abstract dataset. These cases covered five TCM-advantage disease cohorts, two payment pathways, and both pre-policy and post-policy periods. The sample size supported propensity score matching, difference-in-differences analysis, disease-stratified analysis, and sensitivity testing.

The patient-reported component included 545 valid questionnaires. Patients were sampled from the eligible inpatient population using stratified sampling by disease cohort, payment group, age category, and insurance type. The qualitative component included 10 key stakeholders, including patients, TCM clinicians, hospital insurance management staff, and medical insurance administrative personnel.

### 2.6 Eligibility criteria

For the administrative dataset, inclusion criteria were: age ≥18 years, inpatient admission during the study period, principal diagnosis belonging to one of the five selected TCM-advantage disease cohorts, identifiable payment pathway, complete insurance settlement information, complete discharge abstract data, and valid ICD/TCD coding. For the questionnaire dataset, patients also had to provide informed consent and complete the survey within the defined follow-up window.

Exclusion criteria were missing key settlement variables, inconsistent diagnosis-payment matching, unverifiable extreme expenditure, incomplete discharge status, transfer-out records without complete outcome information, terminal malignant disease, severe multi-organ failure unrelated to the target disease, and duplicated short-interval admissions. For patients with repeated admissions, only the first eligible admission was retained in the main analysis; repeated records were assessed in sensitivity analysis.

### 2.7 Exposure and covariates

The primary exposure was settlement under the outcome-based payment pathway for TCM-advantage diseases after policy implementation. Patients were coded as the intervention group if their hospitalization belonged to the designated TCM-advantage disease outcome-based payment pathway. Patients with comparable diagnoses but conventional DRG/DIP settlement were coded as the comparison group.

Covariates included age, sex, insurance type, disease cohort, admission route, hospital level, comorbidity burden, procedure status, baseline severity indicators, pre-policy expenditure level, and year of admission. Comorbidity burden was measured using the Charlson Comorbidity Index when diagnosis coding allowed reliable calculation. Hospital and disease fixed effects were included to reduce institutional and disease-level confounding.

### 2.8 Outcomes and patient-centered public value index

The primary outcome was the Patient-Centered Public Value Index (PCPVI), organized into five domains: health benefit, financial protection, care quality and safety, TCM service provision, and patient experience. The framework links health benefit to symptom and functional measures and EQ-5D-5L; financial protection to out-of-pocket expenditure and perceived burden; care quality and safety to hospital use, discharge status, complications and 30-day readmission; TCM provision to treatment cost proportion and pathway measures; and patient experience to satisfaction, communication and perceived insurance benefit. These domains represent patient-centered evidence relevant to public value, rather than a comprehensive measure of societal value. Financial and utilization measures concern the index hospitalization, questionnaires were administered at discharge, and readmission was assessed within 30 days after discharge.

TCM treatment cost proportion and pathway adherence were interpreted as service-use and care-process proxies. A higher cost share may reflect changes in service volume, prices or other expenditure components and does not establish therapeutic benefit. Syndrome information in medical records was not equivalent to a standardized, validated pre–post syndrome score. The reported analyses therefore do not establish pattern-specific efficacy. Symptom and functional outcomes in the questionnaire were considered separately from TCM-specific syndrome outcomes.

All component indicators were standardized to a 0–100 scale. Positive indicators were directly transformed, whereas negative indicators were reverse coded. The relative importance of these dimensions was evaluated through a two-round modified Delphi consensus process. A purposive panel of 15 experts—comprising five health economists, five senior TCM clinicians, and five medical insurance administrators, each with over 10 years of domain expertise—was convened. All 15 experts responded in both rounds (100% response in each round). In the first round, experts independently rated the importance of each dimension using a 9-point Likert scale. Anonymized statistical feedback, including group medians and interquartile ranges, was circulated prior to the second round to facilitate opinion convergence. Consensus was defined *a priori* as a coefficient of variation (CV) ≤ 0.25 and a Kendall’s coefficient of concordance (W) > 0.5. Kendall’s W was 0.612 in round 1 and 0.738 in round 2 (both *p* < 0.001), and the round 2 CVs ranged from 0.112 to 0.187. After the second round, the finalized mean importance scores were mathematically normalized to 100% to derive the prespecified proportional weights: 30% for health benefit, 20% for financial protection, 20% for care quality and safety, 15% for TCM-specific value, and 15% for patient experience. Health benefit was assigned the highest weight to align with the core objective of healthcare, while financial protection and safety were equally prioritized to reflect the dual goals of insurance reform. Secondary outcomes included total hospitalization expenditure, insurance fund payment, out-of-pocket payment, reimbursement ratio, length of stay, 30-day readmission, TCM treatment cost proportion, EQ-5D-5L score, symptom improvement, and overall satisfaction. Internal consistency was assessed across the aggregated domains. Cronbach’s alpha was 0.82 and ranged from 0.774 to 0.829 after omission of one domain at a time. These statistics alone do not establish the validity or clinical interpretability of the composite index.

### 2.9 Questionnaire and interview procedures

The questionnaire collected demographic information, disease-related recovery, EQ-5D-5L, perceived financial burden, perceived insurance benefit, TCM treatment acceptability, medical communication, and overall satisfaction. The questionnaires were administered face-to-face by trained research assistants on the day of hospital discharge to ensure accurate recall of the inpatient experience. The full survey instrument is provided in Supplementary Appendix S1. EQ-5D-5L assessed health-related quality of life, and disease-specific symptom or functional items were selected for each cohort. Questionnaires with more than 20% missing items or contradictory key responses were excluded.

Semi-structured interviews were conducted with 10 stakeholders (comprising 3 patients, 3 TCM clinicians, 2 hospital insurance management staff, and 2 medical insurance administrative personnel) to contextualize the quantitative findings. Recruitment continued until theoretical saturation was achieved, at which point no new major themes emerged from the concurrent data analysis. The interview guide focused on perceived cost burden, perceived therapeutic value of TCM, influence of payment reform on clinical behavior, coding and pathway implementation, and barriers to public value realization. The semi-structured interview framework is available in Supplementary Appendix S2. Interviews lasted 30–45 min (median, 37 min). Interviews were audio-recorded with consent, transcribed verbatim, and analyzed using thematic analysis. Two researchers independently coded the transcripts; coding agreement was 0.876 and Cohen’s kappa was 0.81. Discrepancies were resolved through discussion.

### 2.10 Statistical analysis

Baseline characteristics were summarized using mean and standard deviation, median and interquartile range, or frequency and percentage. Between-group comparisons used t tests, Mann–Whitney U tests, chi-square tests, or Fisher’s exact tests as appropriate. Propensity score matching was performed to balance baseline differences between payment groups. Specifically, a 1:1 nearest-neighbor matching algorithm without replacement was applied globally across the entire cohort. A tight caliper width set to 0.02 standard deviations of the logit of the propensity score was utilized to ensure optimal covariate balance. Matching variables included age, sex, insurance type, disease cohort, comorbidity burden, hospital level, admission route, procedure status, and baseline severity. Balance was evaluated using standardized mean differences (SMDs), with values below 0.10 indicating acceptable balance.

The main policy effect was estimated using a difference-in-differences model after matching. The interaction term between payment group and post-policy period represented the net effect of outcome-based payment. Linear regression was used for continuous outcomes, generalized linear models with a log link and gamma distribution were used for cost outcomes, and logistic regression was used for binary outcomes. Subgroup analyses were conducted by disease cohort, age group, insurance type, hospital level, comorbidity burden, and TCM treatment intensity. The utilization intensity was operationalized using the financial cost share of traditional therapies within the total hospitalization expenditure. Specifically, cases exceeding the cohort median value were designated as high intensity. Those at or below the median formed the low intensity reference group. Robustness was tested using inverse probability weighting, exclusion of extreme cost records, alternative index weighting, placebo policy timing, and repeated-admission sensitivity analysis. A SHAP-analogous contribution display was used to describe components of PCPVI. This descriptive display was not interpreted as a causal analysis of independent determinants. Statistical analyses were performed using R version 4.3.2 and Stata version 17.0, and qualitative analysis was managed using NVivo version 12.

## 3 Results

### 3.1 Cohort construction and multi-source data integration

Figure 1A illustrates the multi-source data architecture. Medical insurance settlement records, hospital discharge abstracts, electronic medical records, and patient questionnaires were integrated through encrypted patient linkage, ICD/TCD standardization, and data quality filtering. The administrative dataset contained 7,985 inpatient cases, while the patient-reported dataset contained 545 valid questionnaires.

**Figure 1:**
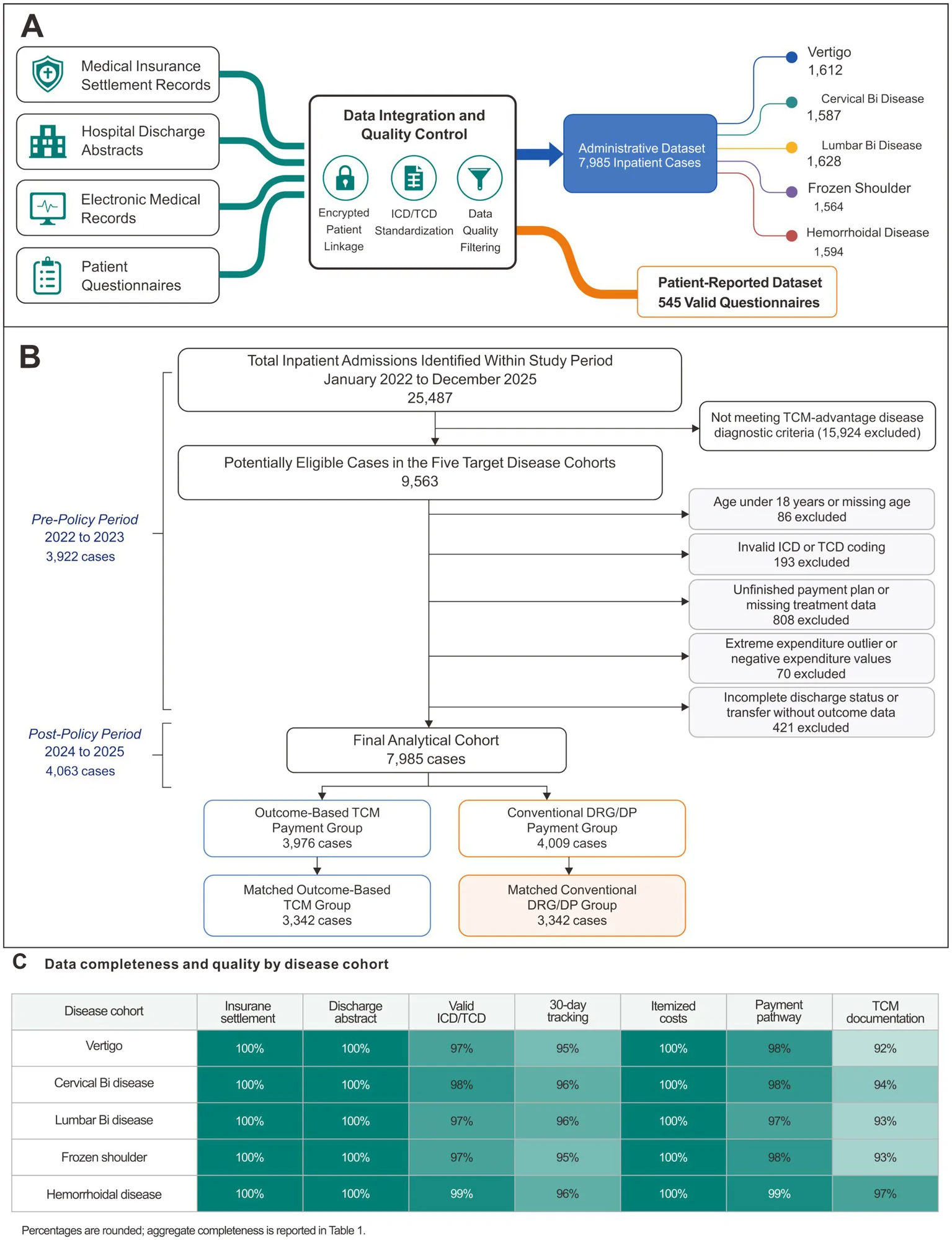
Cohort construction and multi-source data integration. **(A)** Data sources and linkage framework. **(B)** Admissions, exclusions, final cohort and matched group sizes. **(C)** Rounded cohort-level completeness percentages. Aggregate completeness is reported in Table 1. The 545 questionnaire respondents constitute the patient-reported component.

Figure 1B presents cohort selection. Of 25,487 inpatient admissions identified between January 2022 and December 2025, 15,924 were outside the five target disease cohorts, leaving 9,563 potentially eligible admissions. A further 1,578 admissions were excluded, leaving 7,985 cases for analysis. Propensity score matching retained 3,342 cases in each payment group.

Figure 1C displays the data-completeness matrix. Insurance settlement records, discharge abstracts and itemized cost data were complete, while TCM treatment documentation was less complete. The latter limits evaluation of TCM-specific processes and outcomes. Overall cohort completeness for coding linkage and 30-day readmission tracking is reported in Table 1. Complete 30-day follow-up was available for 7,631 of 7,985 cases (95.6%) overall and 6,390 of 6,684 matched cases (95.6%); 294 matched cases lacked complete follow-up.

**Table 1:** Cohort structure and data completeness of the study population.

Variable	Overall, *n* (%)	Outcome-based TCM payment, *n* (%)	Conventional DRG/DIP payment, *n* (%)
Total inpatient cases	7,985 (100.0)	3,976 (49.8)	4,009 (50.2)
Pre-policy period	3,922 (49.1)	1,949 (49.0)	1,973 (49.2)
Post-policy period	4,063 (50.9)	2,027 (51.0)	2,036 (50.8)
Vertigo cohort	1,612 (20.2)	804 (20.2)	808 (20.2)
Cervical Bi disease cohort	1,587 (19.9)	789 (19.8)	798 (19.9)
Lumbar Bi disease cohort	1,628 (20.4)	817 (20.5)	811 (20.2)
Frozen shoulder cohort	1,564 (19.6)	778 (19.6)	786 (19.6)
Hemorrhoidal disease cohort	1,594 (20.0)	788 (19.8)	806 (20.1)
Tertiary hospital	4,545 (56.9)	2,112 (53.1)	2,433 (60.7)
Secondary hospital	3,440 (43.1)	1,864 (46.9)	1,576 (39.3)
Urban employee basic medical insurance	4,551 (57.0)	2,219 (55.8)	2,332 (58.2)
Urban–rural resident basic medical insurance	3,434 (43.0)	1,757 (44.2)	1,677 (41.8)
Complete insurance settlement data	7,985 (100.0)	3,976 (100.0)	4,009 (100.0)
Complete discharge abstract data	7,985 (100.0)	3,976 (100.0)	4,009 (100.0)
Valid ICD–TCD mapping	7,792 (97.6)	3,889 (97.8)	3,903 (97.4)
Complete 30-day readmission tracking	7,631 (95.6)	3,811 (95.8)	3,820 (95.3)
Complete itemized cost structure	7,985 (100.0)	3,976 (100.0)	4,009 (100.0)

Valid ICD-TCD mapping denotes record-level mapping completeness within the final cohort; the disease-cohort eligibility criterion concerns correspondence between diagnostic categories.

Table 1 summarizes the final analytical cohort and data completeness. A total of 7,985 inpatient cases were included, comprising 3,976 cases in the outcome-based TCM payment group and 4,009 cases in the conventional DRG/DIP payment group. The pre-policy and post-policy periods contributed 3,922 and 4,063 cases, respectively. The five disease cohorts were evenly represented, with 1,612 cases of vertigo, 1,587 cases of cervical Bi disease, 1,628 cases of lumbar Bi disease, 1,564 cases of frozen shoulder, and 1,594 cases of hemorrhoidal disease. Insurance settlement data, discharge abstracts, and itemized cost structures were complete for all included cases, while valid ICD–TCD mapping and 30-day readmission tracking were available for 97.6 and 95.6% of cases, respectively.

### 3.2 Baseline characteristics and post-matching balance

Table 2 reports baseline characteristics before and after propensity score matching. Before matching, several covariates showed meaningful imbalance, particularly tertiary hospital admission, procedure or operation status, baseline severity score, and length of stay. After matching, both groups contained 3,342 cases, and all standardized mean differences were reduced below 0.10. The largest residual imbalance after matching was observed for baseline severity score, but the SMD remained low at 0.031.

**Table 2:** Baseline characteristics before and after propensity score matching.

Variable	Before: TCM (*n* = 3,976)	Before: DRG/DIP (*n* = 4,009)	SMD	After: TCM (*n* = 3,342)	After: DRG/DIP (*n* = 3,342)	SMD
Age, years	58.6 ± 14.3	59.8 ± 14.6	0.083	59.0 ± 14.1	59.2 ± 14.3	0.014
Male sex, *n* (%)	1,857 (46.7)	1,996 (49.8)	0.062	1,614 (48.3)	1,607 (48.1)	0.004
Urban employee insurance, *n* (%)	2,219 (55.8)	2,332 (58.2)	0.048	1,905 (57.0)	1,912 (57.2)	0.004
Tertiary hospital, *n* (%)	2,112 (53.1)	2,433 (60.7)	0.153	1,888 (56.5)	1,893 (56.6)	0.002
Charlson Comorbidity Index ≥2, *n* (%)	1,137 (28.6)	1,302 (32.5)	0.085	1,021 (30.6)	1,045 (31.3)	0.015
Emergency admission, *n* (%)	720 (18.1)	862 (21.5)	0.086	679 (20.3)	686 (20.5)	0.005
Procedure or operation, *n* (%)	946 (23.8)	1,135 (28.3)	0.103	876 (26.2)	884 (26.5)	0.007
Baseline severity score	1.73 ± 0.64	1.82 ± 0.67	0.137	1.79 ± 0.64	1.81 ± 0.66	0.031
Length of stay, days	8.1 ± 3.6	8.5 ± 3.8	0.108	8.3 ± 3.7	8.4 ± 3.6	0.027
Total hospitalization expenditure, RMB	10,730 ± 4,860	11,210 ± 5,070	0.097	10,920 ± 4,920	11,010 ± 4,970	0.018
Vertigo cohort, *n* (%)	804 (20.2)	808 (20.2)	0	681 (20.4)	674 (20.2)	0.005
Cervical Bi disease cohort, *n* (%)	789 (19.8)	798 (19.9)	0.002	663 (19.8)	669 (20.0)	0.005
Lumbar Bi disease cohort, *n* (%)	817 (20.5)	811 (20.2)	0.007	688 (20.6)	685 (20.5)	0.002
Frozen shoulder cohort, *n* (%)	778 (19.6)	786 (19.6)	0	650 (19.4)	654 (19.6)	0.005
Hemorrhoidal disease cohort, *n* (%)	788 (19.8)	806 (20.1)	0.007	660 (19.7)	660 (19.7)	0.000

Figure 2A confirms the improvement in covariate balance. Before matching, several covariates approached or exceeded the 0.10 SMD threshold, including tertiary hospital, procedure or operation, baseline severity score, and length of stay. After matching, all covariates clustered near zero, supporting adequate comparability between groups.

**Figure 2:**
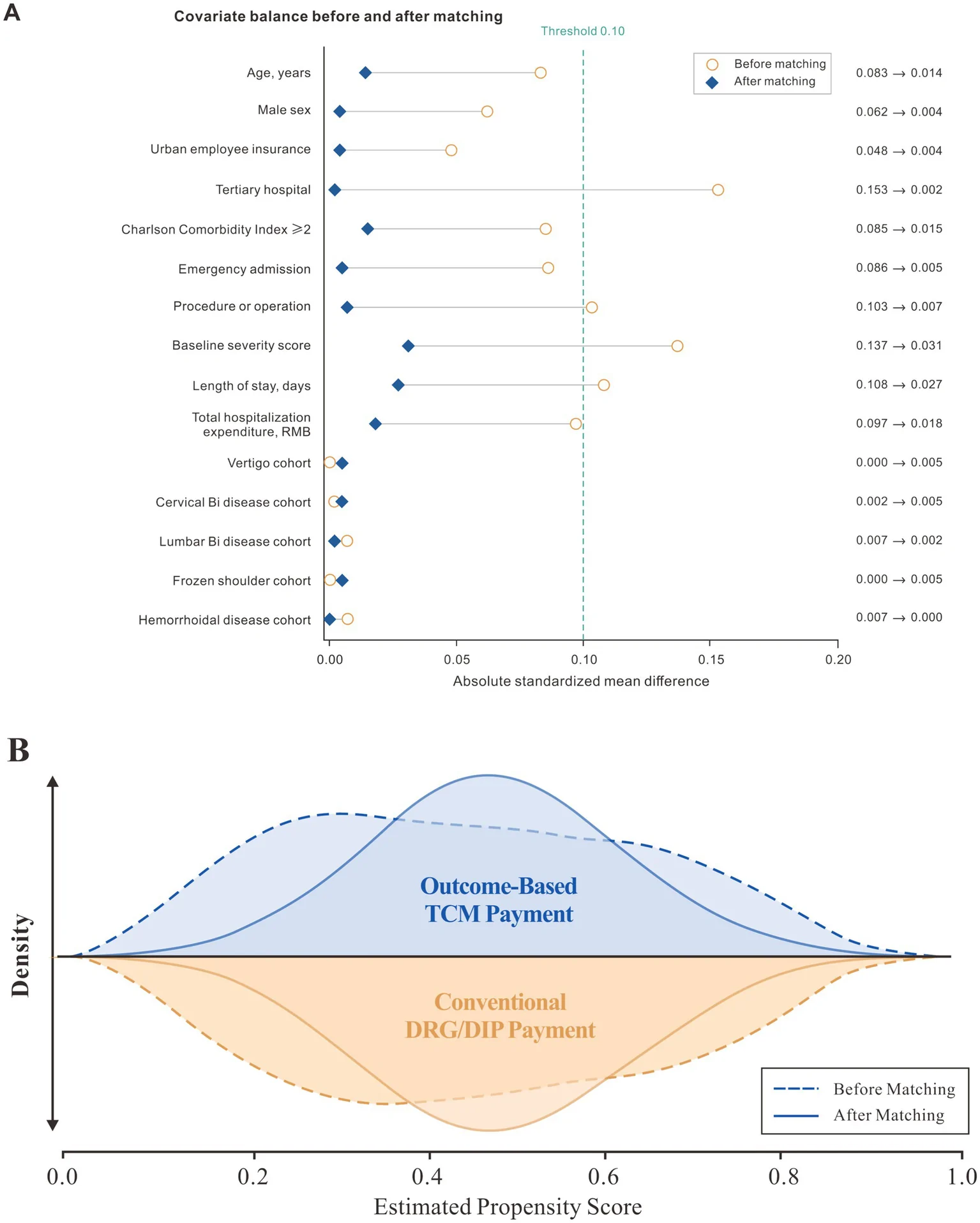
Covariate balance before and after propensity score matching. **(A)** Absolute standardized mean differences for all 15 covariates in Table 2. Circles and diamonds denote before and after matching; the dashed line marks 0.10. **(B)** Propensity score distributions before and after matching.

Figure 2B displays the propensity score distributions before and after matching. The curves suggest greater overlap after matching; covariate balance is assessed quantitatively using the individual SMDs in Figure 2A and Table 2.

### 3.3 Descriptive changes in insurance payment, resource use, and care quality

Table 3 presents unadjusted pre-post changes after matching. In the conventional DRG/DIP group, total hospitalization expenditure increased from 10,860 RMB to 11,180 RMB, and out-of-pocket payment increased from 3,280 RMB to 3,370 RMB. In contrast, the outcome-based TCM payment group showed decreases in total hospitalization expenditure from 10,740 RMB to 10,540 RMB and out-of-pocket payment from 3,260 RMB to 3,050 RMB. The reimbursement ratio increased more clearly in the outcome-based TCM group than in the conventional group, rising from 68.9 to 71.6%. The TCM treatment cost proportion increased from 14.1 to 19.3% in the outcome-based TCM group, compared with only a modest increase from 13.4 to 13.7% in the conventional group.

**Table 3:** Descriptive comparison of cost, utilization, and quality outcomes by payment group and policy period.

Outcome	DRG/DIP pre (*n* = 1,657)	DRG/DIP post (*n* = 1,685)	Change	TCM pre (*n* = 1,642)	TCM post (*n* = 1,700)	Change
Total hospitalization expenditure, RMB	10,860 (8,520–14,180)	11,180 (8,760–14,640)	320	10,740 (8,420–13,950)	10,540 (8,190–13,610)	−200
Out-of-pocket payment, RMB	3,280 (2,130–4,720)	3,370 (2,220–4,880)	90	3,260 (2,100–4,660)	3,050 (1,950–4,380)	−210
Insurance fund payment, RMB	6,740 (5,010–9,080)	6,950 (5,180–9,340)	210	6,660 (4,920–8,970)	6,740 (5,020–9,160)	80
Reimbursement ratio, %	68.7 ± 12.3	69.0 ± 12.1	0.3	68.9 ± 12.2	71.6 ± 11.8	2.7 pp
Length of stay, days	8.4 ± 3.7	8.3 ± 3.6	−0.1	8.3 ± 3.6	7.9 ± 3.4	−0.4
30-day readmission, *n* (%)	122 (7.4)	120 (7.1)	−0.3 pp	119 (7.2)	114 (6.7)	−0.5 pp
Complications during hospitalization, *n* (%)	96 (5.8)	98 (5.8)	0.0 pp	92 (5.6)	89 (5.2)	−0.4 pp
TCM treatment cost proportion, %	13.4 ± 6.5	13.7 ± 6.6	0.3 pp	14.1 ± 6.8	19.3 ± 7.9	5.2
Patient-Centered Public Value Index	63.1 ± 10.4	64.7 ± 10.2	1.6	63.4 ± 10.5	69.9 ± 10.1	6.5

Column sample sizes describe the matched cohort. Complete 30-day follow-up was available for 6,390 of 6,684 cases overall (95.6%). Expenditure values are medians (interquartile ranges); other continuous variables are means +/– standard deviations. Event outcomes are presented as counts and percentages. Change denotes post-policy minus pre-policy. Changes in reimbursement ratio, TCM treatment cost proportion and event percentages are expressed in percentage points (pp).

Figure 3 summarizes the reported changes in composite, financial, service-use and clinical outcomes. Estimates for insurance fund payment, 30-day readmission, complications and EQ-5D-5L were not statistically significant. Outcomes with different units should be interpreted separately using the estimates and confidence intervals in Table 4.

**Figure 3:**
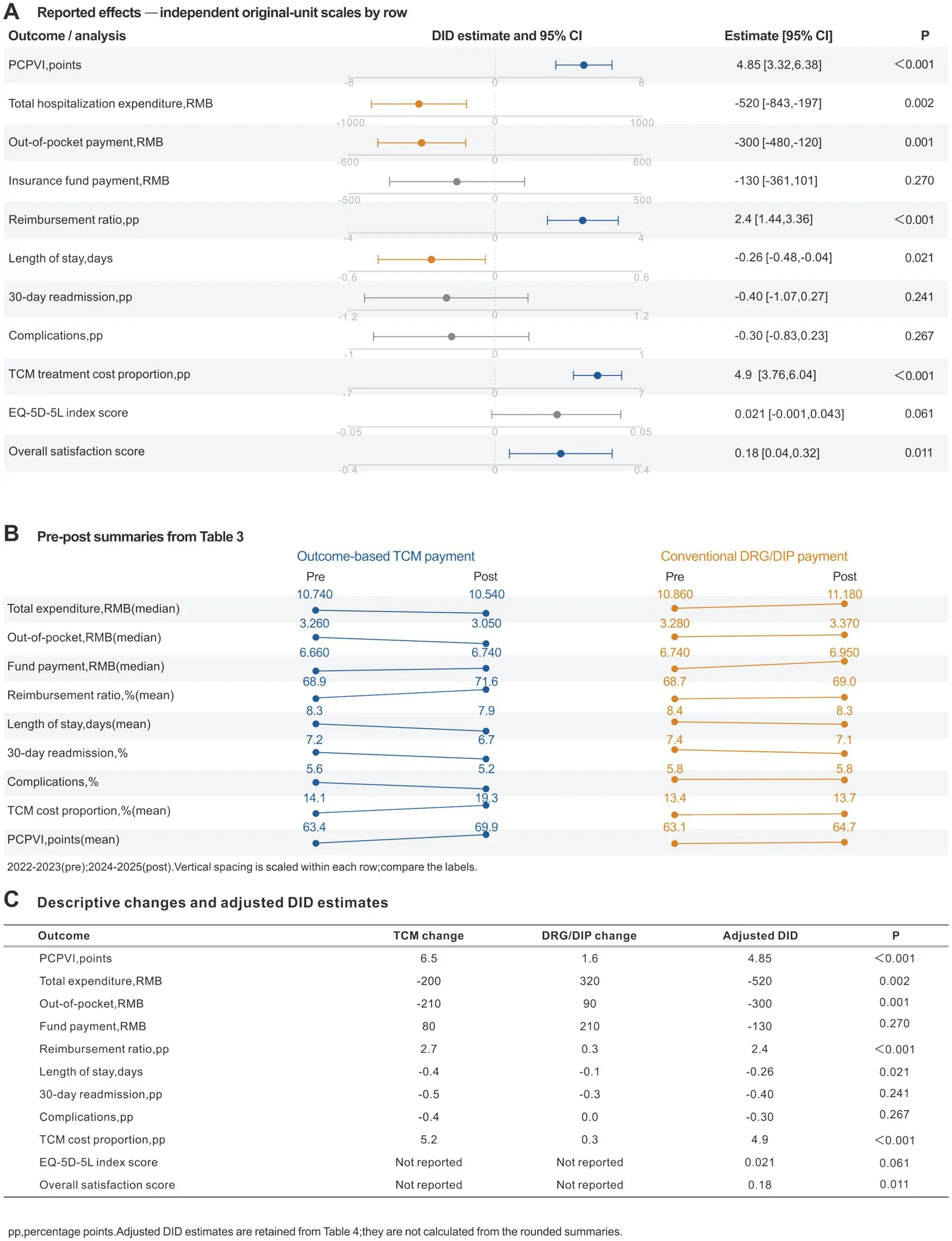
Pre–post summaries and reported difference-in-differences effects. **(A)** Points and horizontal bars show Table 4 DID estimates and 95% confidence intervals. Each row uses its own labelled axis in the outcome’s original units; horizontal distances are not comparable across rows. Grey marks denote *p* ≥ 0.05. **(B)** Table 3 expenditure medians, other continuous-outcome means and event percentages for 2022–2023 and 2024–2025; lines show change on within-row scales. **(C)** Descriptive changes alongside adjusted DID estimates, which are not computed from the rounded summaries. pp., percentage points.

**Table 4:** Difference-in-differences estimates for insurance, clinical, and TCM-specific outcomes.

Outcome	DID estimate	Standard error	95% CI	*p* value
Patient-Centered Public Value Index	4.85	0.78	3.32 to 6.38	<0.001
Total hospitalization expenditure, RMB	−520	165	−843 to −197	0.002
Out-of-pocket payment, RMB	−300	92	−480 to −120	0.001
Insurance fund payment, RMB	−130	118	−361 to 101	0.270
Reimbursement ratio, percentage points	2.4	0.49	1.44 to 3.36	<0.001
Length of stay, days	−0.26	0.11	−0.48 to −0.04	0.021
30-day readmission, percentage points	−0.40	0.34	−1.07 to 0.27	0.241
Complications, percentage points	−0.30	0.27	−0.83 to 0.23	0.267
TCM treatment cost proportion, percentage points	4.9	0.58	3.76 to 6.04	<0.001
EQ-5D-5L index score	0.021	0.011	−0.001 to 0.043	0.061
Overall satisfaction score	0.18	0.07	0.04 to 0.32	0.011

DID, difference-in-differences. Estimates, standard errors, and 95% confidence intervals are reported in the units specified for each outcome. Cost outcomes are expressed as absolute differences in RMB. Reimbursement ratio, 30-day readmission, complications, and TCM treatment cost proportion are expressed as differences in percentage points. Length of stay is expressed in days. The Patient-Centered Public Value Index, EQ-5D-5L index, and overall satisfaction are expressed in their respective score units. Cost and binary-outcome estimates are marginal DID contrasts on the outcome scale, rather than untransformed log-link or log-odds interaction coefficients.

Figure 3B visualizes the pre–post summaries in Table 3: medians for expenditure, means for reimbursement ratio, length of stay, TCM expenditure share and PCPVI, and percentages for readmission and complications. The outcome-based TCM group showed lower total expenditure, out-of-pocket payment, length of stay, readmission and complications, together with higher reimbursement ratio, TCM expenditure share and PCPVI. Connecting lines show within-row change; their slopes are not comparable across outcomes.

Figure 3C summarizes descriptive changes alongside adjusted DID estimates. The increased TCM expenditure share describes a shift in service composition. It does not establish greater TCM-specific clinical efficacy, and descriptive pre–post differences should not be interpreted as adjusted causal effects.

### 3.4 Main policy effect of outcome-based payment

The adjusted DID estimate for PCPVI was 4.85 points (95% CI: 3.32 to 6.38; *p* < 0.001). Total hospitalization expenditure decreased by 520 RMB (95% CI: −843 to −197; *p* = 0.002), and out-of-pocket payment decreased by 300 RMB (95% CI: −480 to −120; *p* = 0.001). The insurance fund payment estimate was −130 RMB (95% CI: −361 to 101; *p* = 0.270), with no statistically significant difference. The reimbursement ratio increased by 2.40 percentage points (*p* < 0.001), and length of stay decreased by 0.26 days (*p* = 0.021). The estimates for 30-day readmission (−0.40 percentage points; 95% CI: −1.07 to 0.27) and complications (−0.30 percentage points; 95% CI: −0.83 to 0.23) were not statistically significant. TCM treatment cost proportion increased by 4.90 percentage points (*p* < 0.001). The EQ-5D-5L estimate was 0.021 (95% CI: −0.001 to 0.043; *p* = 0.061), whereas the satisfaction estimate was 0.18 (95% CI: 0.04 to 0.32; *p* = 0.011).

Figure 4A summarizes the main DID estimates and sensitivity analyses. The numerical estimates and confidence intervals should be read in their original units. Non-significant results for insurance fund payment, readmission, complications and EQ-5D-5L do not establish equivalence, non-inferiority or absence of clinically relevant effects.

**Figure 4:**
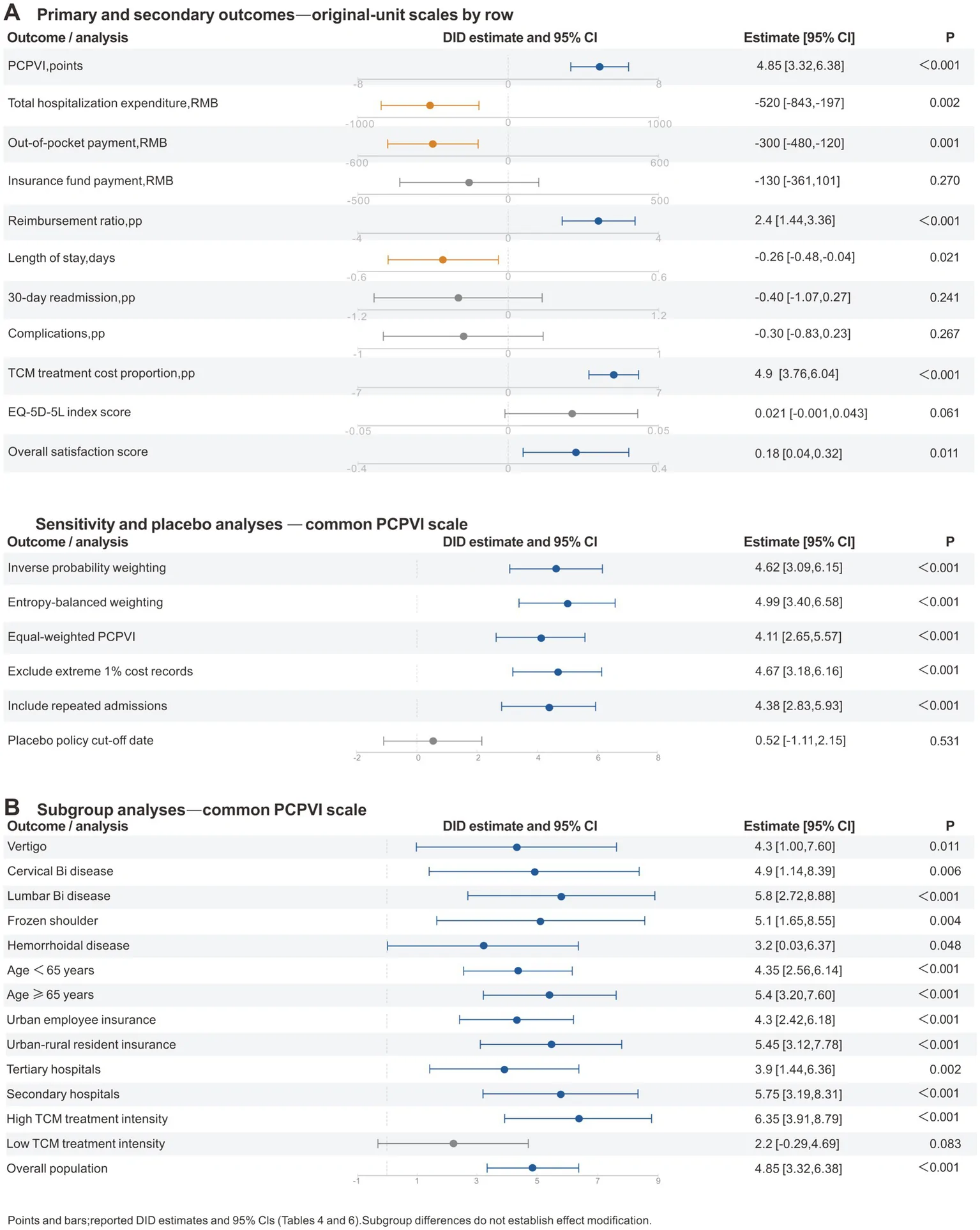
Main, Sensitivity and Subgroup Estimates. **(A)** Main outcome estimates from Table 4 use independent, labelled original-unit axes; sensitivity and placebo estimates from Table 6 share a PCPVI-point scale. **(B)** Subgroup and overall PCPVI estimates from Table 6 share a common scale. Points and bars represent reported DID estimates and 95% confidence intervals; grey marks denote *p* ≥ 0.05. Significance within individual subgroups does not establish differences between subgroups.

The PCPVI estimate remained positive under the sensitivity specifications. The reported pre-policy index coefficient was 0.15 (*p* = 0.623); the joint event-study test of pre-policy coefficients yielded *p* = 0.482, and the placebo estimate was 0.52 (95% CI: −1.11 to 2.15; *p* = 0.531). These results did not detect a pre-policy difference or placebo effect, but failure to reject a null hypothesis does not prove parallel trends or eliminate time-varying confounding.

Figure 4B presents subgroup effects for the Patient-Centered Public Value Index. Positive effects were observed across all five disease cohorts, both age groups, both insurance types, and both hospital levels. The largest disease-specific effect was observed in lumbar Bi disease, while the smallest was observed in hemorrhoidal disease. The effect was stronger among patients with high TCM treatment intensity, whereas the low TCM treatment intensity subgroup showed a smaller and non-significant effect, but these descriptive differences do not establish effect modification.

### 3.5 Patient-reported outcomes and perceived insurance benefit

Among 545 questionnaire respondents, 276 were in the outcome-based TCM payment group and 269 were in the conventional DRG/DIP group (Table 5). Age, sex, and insurance type were comparable between groups. The outcome-based TCM payment group reported a higher EQ visual analogue scale (EQ-VAS) score than the conventional group (76.6 vs. 74.0, *p* = 0.035). Conversely, the difference observed in the EQ-5D-5L index score (0.85 vs. 0.83) was not statistically significant (*p* = 0.071). Clinically meaningful symptom improvement, good functional recovery, lower perceived financial burden, and willingness to choose the TCM pathway again all favored the outcome-based TCM group, although none of these categorical differences achieved statistical significance.

**Table 5:** Patient-reported outcomes, satisfaction, and insurance benefit perception among 545 respondents.

Variable	Overall (*n* = 545)	Outcome-based TCM payment (*n* = 276)	Conventional DRG/DIP payment (*n* = 269)	*p* value
Age, years	58.8 ± 13.7	58.3 ± 13.5	59.3 ± 13.9	0.456
Male sex, *n* (%)	263 (48.3)	132 (47.8)	131 (48.7)	0.795
Urban employee insurance, *n* (%)	309 (56.7)	153 (55.4)	156 (58.0)	0.541
EQ-5D-5L index score	0.84 ± 0.14	0.85 ± 0.13	0.83 ± 0.14	0.071
EQ-VAS score	75.3 ± 14.4	76.6 ± 13.9	74.0 ± 14.7	0.035
Clinically meaningful symptom improvement, *n* (%)	364 (66.8)	194 (70.3)	170 (63.2)	0.078
Good functional recovery, *n* (%)	341 (62.6)	183 (66.3)	158 (58.7)	0.064
Pain or discomfort improved by ≥2 points, *n* (%)	326 (59.8)	174 (63.0)	152 (56.5)	0.121
Low-to-moderate perceived financial burden, *n* (%)	350 (64.2)	188 (68.1)	162 (60.2)	0.055
Insurance benefit perception score	3.96 ± 0.75	4.06 ± 0.72	3.86 ± 0.75	0.002
Communication quality score	4.10 ± 0.68	4.18 ± 0.65	4.02 ± 0.70	0.008
TCM treatment acceptability score	4.10 ± 0.71	4.25 ± 0.63	3.95 ± 0.76	<0.001
Overall satisfaction score	4.11 ± 0.70	4.20 ± 0.66	4.02 ± 0.73	0.003
Willingness to choose the TCM pathway again, *n* (%)	401 (73.6)	212 (76.8)	187 (69.5)	0.055

Continuous variables are presented as means +/– standard deviations, and categorical variables as *n* (%).

Clearer differences were observed in patient-perceived payment and care experience indicators. The outcome-based TCM payment group had higher insurance benefit perception (4.06 vs. 3.86, *p* = 0.002), communication quality (4.18 vs. 4.02, *p* = 0.008), TCM treatment acceptability (4.25 vs. 3.95, *p* < 0.001), and overall satisfaction (4.20 vs. 4.02, *p* = 0.003). These findings indicate that patient-reported benefits were most evident in perceived insurance value, care experience, and acceptability of TCM treatment.

### 3.6 Patient-centered public value profile and mechanism pathway

Figure 5A displays the five reported PCPVI dimension scores. Arithmetic means of the rounded displayed values were 62.4 and 62.0 before the policy, and 72.4 and 65.4 after the policy, for the outcome-based TCM and conventional DRG/DIP groups, respectively. These are unweighted descriptive summaries, not the weighted PCPVI estimates in Table 3. The largest post-policy separation was in the TCM-specific domain (76 versus 59); health benefit and patient experience each differed by six points, while financial protection and care quality and safety each differed by three points. These domain labels do not establish direct TCM efficacy or clinical safety.

**Figure 5:**
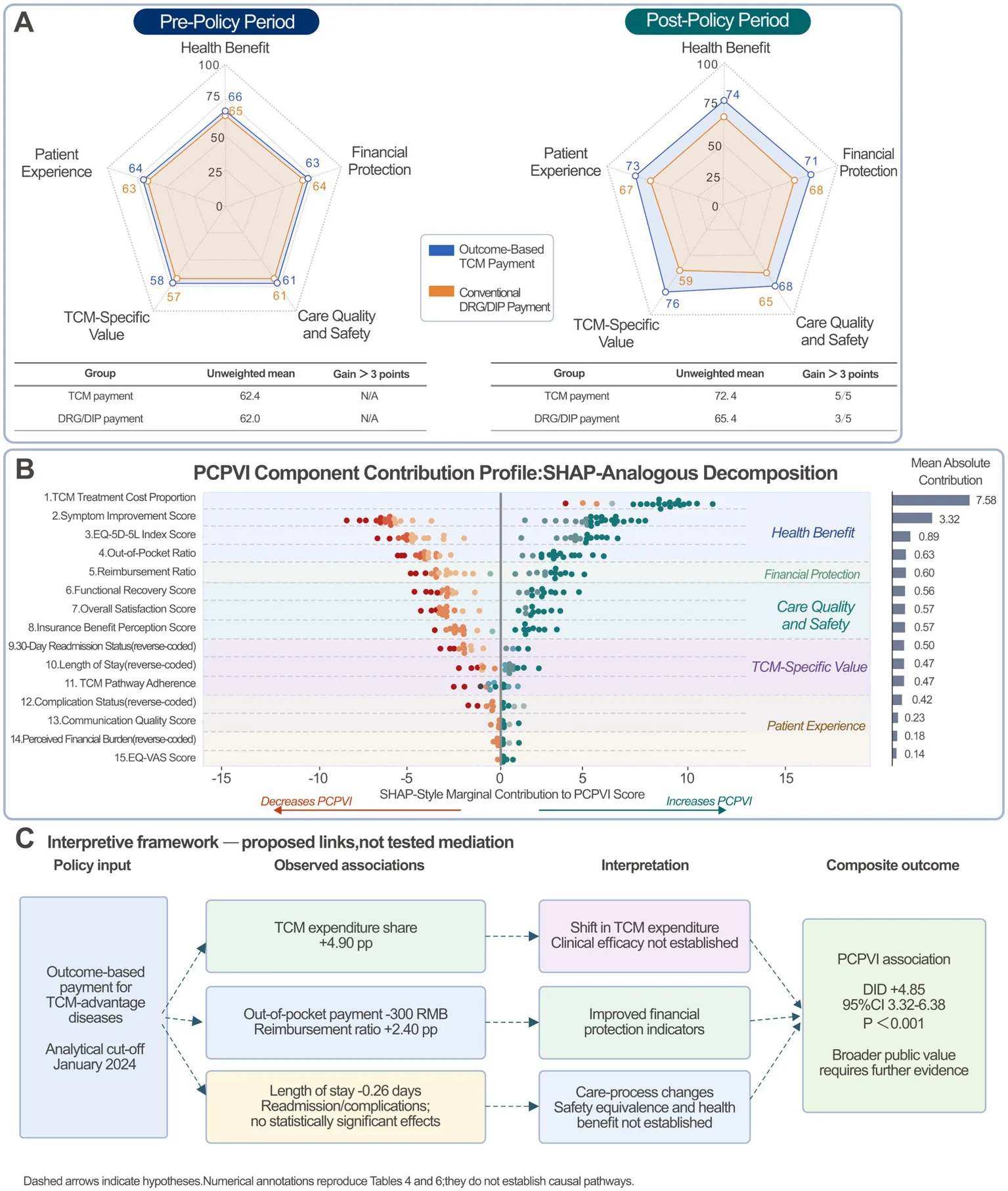
PCPVI domain profiles and interpretive framework. **(A)** Rounded domain scores; the small tables give unweighted arithmetic means and counts of domains gaining strictly more than three points. These summaries are not the weighted PCPVI. **(B)** SHAP-analogous contribution display of PCPVI components. **(C)** Proposed links between payment incentives, observed outcomes and PCPVI. Dashed arrows indicate hypotheses, not tested mediation. Increased TCM expenditure share does not establish TCM-specific clinical efficacy; non-significant safety outcomes do not establish equivalence.

Figure 5B presents a SHAP-analogous component-contribution display. TCM treatment cost proportion has the largest displayed mean absolute contribution, followed by symptom improvement and EQ-5D-5L. Because the display concerns components already included in PCPVI, it does not identify independent causal drivers of public value. The reported sensitivity analysis excluding TCM treatment cost proportion yielded a DID estimate of 3.72 (95% CI: 2.15 to 5.29; *p* < 0.001). Its interpretation depends on the specified index reconstruction and model; the display alone cannot validate that analysis.

Figure 5C organizes proposed mechanisms connecting payment incentives, TCM service provision, financial protection and care processes. The observed increases in TCM expenditure share and reimbursement ratio, and decreases in out-of-pocket payment and length of stay, are consistent with parts of this framework. The diagram is an interpretive model, not a formal mediation analysis or proof that increased TCM provision caused better clinical outcomes.

### 3.7 Subgroup, sensitivity, and qualitative integration analyses

PCPVI estimates were positive across the five disease cohorts, ranging from 3.20 points for hemorrhoidal disease (95% CI: 0.03 to 6.37) to 5.80 points for lumbar Bi disease (95% CI: 2.72 to 8.88) (Table 6). Positive estimates were also reported across age, insurance and hospital-level subgroups. Differences between subgroup point estimates alone do not establish effect modification, and these within-Taiyuan comparisons do not demonstrate applicability to other provinces.

**Table 6:** Subgroup, sensitivity, and placebo analysis results.

Analysis	Subgroup or model	Effect on patient-centered public value index	95% CI	*p* value
Main matched DID model	Overall population	4.85	3.32 to 6.38	<0.001
Subgroup analysis	Vertigo cohort	4.3	1.00 to 7.60	0.011
Subgroup analysis	Cervical Bi disease cohort	4.9	1.41 to 8.39	0.006
Subgroup analysis	Lumbar Bi disease cohort	5.8	2.72 to 8.88	<0.001
Subgroup analysis	Frozen shoulder cohort	5.1	1.65 to 8.55	0.004
Subgroup analysis	Hemorrhoidal disease cohort	3.2	0.03 to 6.37	0.048
Subgroup analysis	Age <65 years	4.35	2.56 to 6.14	<0.001
Subgroup analysis	Age ≥65 years	5.4	3.20 to 7.60	<0.001
Subgroup analysis	Urban employee insurance	4.3	2.42 to 6.18	<0.001
Subgroup analysis	Urban–rural resident insurance	5.45	3.12 to 7.78	<0.001
Subgroup analysis	Tertiary hospitals	3.9	1.44 to 6.36	0.002
Subgroup analysis	Secondary hospitals	5.75	3.19 to 8.31	<0.001
Subgroup analysis	High TCM treatment intensity	6.35	3.91 to 8.79	<0.001
Subgroup analysis	Low TCM treatment intensity	2.2	−0.29 to 4.69	0.083
Sensitivity analysis	Inverse probability weighting	4.62	3.09 to 6.15	<0.001
Sensitivity analysis	Entropy-balanced weighting	4.99	3.40 to 6.58	<0.001
Sensitivity analysis	Equal-weighted public value index	4.11	2.65 to 5.57	<0.001
Sensitivity analysis	Exclusion of top and bottom 1% cost records	4.67	3.18 to 6.16	<0.001
Sensitivity analysis	Inclusion of repeated admissions	4.38	2.83 to 5.93	<0.001
Placebo test	False policy cut-off date	0.52	−1.11 to 2.15	0.531

The PCPVI estimate was 6.35 points in the high TCM treatment intensity subgroup (95% CI: 3.91 to 8.79) and 2.20 points in the low intensity subgroup (95% CI: −0.29 to 4.69). These are exploratory comparisons. Intensity was defined by the TCM expenditure share, which may itself be affected by payment reform and contributes to the composite index. The contrast therefore cannot establish a dose–response relationship or the therapeutic effect of greater TCM use.

The reported sensitivity analyses yielded estimates consistent in direction with the main finding. The PCPVI effect remained significant under inverse probability weighting, entropy-balanced weighting, equal-weighted index construction, exclusion of extreme cost records, and inclusion of repeated admissions. The placebo test was non-significant, but does not establish the validity of the policy timing. Qualitative integration, wherein 10 stakeholders were interviewed, provided context for the quantitative findings: reduced perceived out-of-pocket burden, improved recognition of TCM pathway value, and more standardized clinical pathway management were commonly reported. However, coding and documentation burden and the need for dynamic payment-standard adjustment were also identified as implementation challenges (Table 7).

**Table 7:** Qualitative findings from 10 stakeholder interviews.

Theme	Supporting interviews, *n*/10	Direction of interpretation
Reduced perceived out-of-pocket burden	6/10	Consistent with lower out-of-pocket payment
Improved recognition of TCM pathway value	7/10	Consistent with increased TCM service use and perceived pathway value
More standardized clinical pathway management	5/10	Partly supported care process improvement
Increased coding and documentation burden	5/10	Explained implementation workload
Need for dynamic adjustment of payment standards	7/10	Suggested future payment refinement
Concern about hospital cost-control pressure	4/10	Indicated potential implementation risk

## 4 Discussion

Outcome-based payment in Taiyuan was associated with a 4.85-point higher PCPVI, lower patient expenditure and more favorable care experience. These findings are relevant to patient-centered public value, but the index captures only part of the wider public-management concept. Neither its minimum important difference nor its external validity has been established. The results therefore support a cautious interpretation of improved measured patient benefit, rather than a claim of demonstrated societal value, clinical superiority or fund sustainability.

Lower total hospitalization expenditure and out-of-pocket payment, together with a higher reimbursement ratio, suggest improved financial protection during the index admission. The insurance fund payment estimate was −130 RMB, but its 95% confidence interval ranged from a reduction of 361 RMB to an increase of 101 RMB per admission (*p* = 0.270). The data therefore provide no statistically conclusive evidence of a change in fund payment. The negative point estimate should not be described as a savings trend. Nor do these findings establish budget neutrality or long-term sustainability: admission volume, subsequent care, administrative costs and longer-term outcomes were not captured by the per-admission estimate. A formal cost-effectiveness or budget-impact analysis would be required before calling the policy economically dominant.

The 4.90-percentage-point increase in TCM treatment cost proportion indicates a change in expenditure composition. It may reflect greater TCM provision, reduced spending on other services or changes in relative prices; the cost share alone cannot distinguish these explanations. Pathway adherence similarly concerns care processes and cannot substitute for clinical outcomes. A separate reporting tradition exists for TCM-specific efficacy: the CONSORT extension for Chinese herbal medicine formulas calls for clearly defined pattern-related outcomes and assessment methods (24). Although that guidance addresses randomized trials rather than payment evaluations, it highlights the need to record the diagnostic pattern, a reproducible syndrome scale and assessment timing. Future payment evaluations should collect standardized baseline and follow-up syndrome scores alongside disease-specific symptoms, function and adverse events. The present proxy measures cannot establish such efficacy.

The EQ-5D-5L estimate of 0.021 was not statistically significant (95% CI: −0.001 to 0.043; *p* = 0.061). This interval is compatible with little difference as well as a positive effect; clinical importance cannot be determined without an appropriate, prespecified threshold. Chronicity and assessment at discharge may influence responsiveness, but neither explains the result by itself. Lam et al. found responsiveness of the electronic EQ-5D-5L over three months among Chinese patients with chronic knee or back problems (25), so chronic disease should not be equated with instrument insensitivity. That population, administration mode and follow-up differ from this study, and its results cannot establish benefit in our cohort. The smaller questionnaire sample, heterogeneous conditions and possible ceiling effects are alternative explanations that were not directly tested. Longer follow-up, paired baseline assessments, examination of ceiling effects and validated disease-specific measures would help distinguish limited sensitivity from a small or absent effect. Higher satisfaction, communication and insurance-benefit scores concern patient experience and should not be presented as substitutes for health improvement.

Transferability depends on the policy and delivery setting. Provincial and local TCM-advantage catalogues determine eligible patients, while DRG weights, DIP points, payment standards and settlement rules shape provider incentives. Hospital capacity, coding quality and the availability of TCM practitioners may further affect implementation. The positive within-Taiyuan subgroup estimates do not resolve these differences. The evaluative framework may inform other settings, but the observed effect sizes and local reimbursement arrangements require validation before use elsewhere. Multiregional studies should report catalogue eligibility, comparator payment rules and implementation timing, and assess whether effects vary across these conditions.

The qualitative findings provide contextual depth. Interviewees described reduced financial burden and greater recognition of TCM pathways, alongside coding burden, documentation workload and the need to adjust payment standards over time. These are perceptions from the present interviews and do not independently verify the quantitative effects. External work on value-based payment identifies implementation capacity, outcome measurement and stakeholder coordination as relevant design considerations (9, 11). Evidence on provider responses to case-based payment also supports monitoring changes in treatment intensity and case mix (2, 18). These considerations favor ongoing assessment of patient access and care quality; they do not establish that cream-skimming or pathway compression occurred in this cohort.

Several limitations affect interpretation. First, matching and DID cannot exclude unmeasured confounding, differential policy changes or violations of parallel trends. Second, the single-city setting limits external validity. Third, the 545-questionnaire component is smaller than the administrative cohort and may be affected by response selection; transparent linkage, outcome-specific denominators and missing-data procedures are essential for interpreting the composite index. In addition, 294 matched cases lacked complete 30-day follow-up, creating potential attrition bias in readmission comparisons. Fourth, incomplete TCM documentation and reliance on service-use proxies limit assessment of syndrome-specific efficacy. Fifth, discharge EQ-5D-5L assessments provide limited information about sustained recovery, and responsiveness and ceiling effects were not directly evaluated. Sixth, coding responses, upcoding and administrative implementation costs were not separately quantified. Finally, the non-significant fund-payment and safety estimates do not demonstrate equivalence or non-inferiority. Longer patient follow-up, transparent composite-index validation and multiregional evaluation are needed to assess clinical benefit, equity and fiscal consequences.

## 5 Conclusion

In Taiyuan, outcome-based payment for TCM-advantage diseases was associated with a higher PCPVI, lower out-of-pocket payment and more favorable patient experience. These findings support further evaluation of payment arrangements that align patient benefit with accountable use of insurance resources. They do not establish TCM-specific therapeutic efficacy, improved EQ-5D-5L outcomes, insurance fund savings or non-inferior safety. The PCPVI requires transparent measurement and external validation, and wider implementation should be informed by regional payment rules and longer-term clinical and financial evidence.

## Data Availability

The raw data supporting the conclusions of this article will be made available by the authors, without undue reservation.
