# Supplementary material for: Aligning insurance payment with public value: outcome-based payment for traditional Chinese medicine advantage diseases under China’s DRG/DIP reform
Source: Front Public Health. 2026 Oct 21;14:1902781. doi: 10.3389/fpubh.2026.1902781 (PMC13730889; doi:10.3389/fpubh.2026.1902781)
Supplement: Supplementary file 1 [file Table_1.DOCX]

**Supplementary Appendix S1**

**Patient Questionnaire for the Evaluation of Patient-Centered Public Value**

**Instructions for Interviewers:** This questionnaire is to be administered face-to-face to eligible patients on the day of hospital discharge. Please ensure the patient understands that their responses will remain confidential and will not affect their medical insurance reimbursement or future treatment.

**Section A: Demographic and Basic Information**

*(To be completed with the patient or verified via electronic medical records)*

**A1. Age:** ______ years old

**A2. Sex:**

[ ] 1. Male

[ ] 2. Female

**A3. Primary Medical Insurance Type:**

[ ] 1. Urban Employee Basic Medical Insurance (UEBMI)

[ ] 2. Urban-Rural Resident Basic Medical Insurance (URRBMI)

[ ] 3. Other: ______________

**A4. Primary Diagnosis (TCM-Advantage Disease Cohort):**

[ ] 1. Vertigo

[ ] 2. Cervical Bi Disease

[ ] 3. Lumbar Bi Disease

[ ] 4. Frozen Shoulder

[ ] 5. Hemorrhoidal Disease

**Section B: Health-Related Quality of Life (EQ-5D-5L & EQ-VAS)**

*(Standard EQ-5D-5L wording is applied here. Please check the box that best describes your health TODAY.)*

**B1. Mobility**

[ ] 1. I have no problems in walking about

[ ] 2. I have slight problems in walking about

[ ] 3. I have moderate problems in walking about

[ ] 4. I have severe problems in walking about

[ ] 5. I am unable to walk about

**B2. Self-Care**

[ ] 1. I have no problems washing or dressing myself

[ ] 2. I have slight problems washing or dressing myself

[ ] 3. I have moderate problems washing or dressing myself

[ ] 4. I have severe problems washing or dressing myself

[ ] 5. I am unable to wash or dress myself

**B3. Usual Activities (e.g., work, study, housework, family or leisure activities)**

[ ] 1. I have no problems doing my usual activities

[ ] 2. I have slight problems doing my usual activities

[ ] 3. I have moderate problems doing my usual activities

[ ] 4. I have severe problems doing my usual activities

[ ] 5. I am unable to do my usual activities

**B4. Pain / Discomfort**

[ ] 1. I have no pain or discomfort

[ ] 2. I have slight pain or discomfort

[ ] 3. I have moderate pain or discomfort

[ ] 4. I have severe pain or discomfort

[ ] 5. I have extreme pain or discomfort

**B5. Anxiety / Depression**

[ ] 1. I am not anxious or depressed

[ ] 2. I am slightly anxious or depressed

[ ] 3. I am moderately anxious or depressed

[ ] 4. I am severely anxious or depressed

[ ] 5. I am extremely anxious or depressed

**B6. Visual Analogue Scale (EQ-VAS)**

We would like to know how good or bad your health is TODAY.

- 100 means the best health you can imagine.
- 0 means the worst health you can imagine.

Please state a number between 0 and 100 to indicate your health today: **[ _______ ]**

**Section C: Disease-Specific Clinical Recovery**

**C1. Compared to when you were admitted, how would you rate your overall symptom improvement?**

[ ] 1. Completely resolved (Excellent improvement)

[ ] 2. Significantly improved (Clinically meaningful improvement)

[ ] 3. Slightly improved

[ ] 4. No change

[ ] 5. Worsened

**C2. How would you rate your functional recovery (ability to perform physical functions related to your specific disease, e.g., joint mobility, daily movement)?**

[ ] 1. Fully recovered

[ ] 2. Good functional recovery

[ ] 3. Moderate functional recovery

[ ] 4. Poor functional recovery / Unchanged

[ ] 5. Worsened

**C3. Please rate your primary pain or discomfort level on a scale of 0 to 10 (0 = no pain, 10 = worst possible pain):**

- Score at the time of **admission**: [ _____ ]
- Score at the time of **discharge (today)**: [ _____ ]

*(Note: A reduction of ≥2 points is defined as a meaningful improvement)*

**Section D: Financial Protection and Insurance Perception**

**D1. Considering your household income, how would you rate the out-of-pocket financial burden of this hospitalization?**

[ ] 1. Very low burden

[ ] 2. Low-to-moderate burden

[ ] 3. Moderate burden

[ ] 4. Heavy burden

[ ] 5. Very heavy burden

**D2. Insurance Benefit Perception**

*Statement: "The medical insurance reimbursement policies for this hospitalization provided me with adequate financial support and reasonable coverage for the treatments I needed."*

[ ] 1. Strongly disagree

[ ] 2. Disagree

[ ] 3. Neutral

[ ] 4. Agree

[ ] 5. Strongly agree

**Section E: Care Experience and TCM Acceptability**

**E1. Communication Quality**

*Statement: "The doctors and nurses clearly communicated my treatment plan, thoroughly explained the purpose of the Traditional Chinese Medicine (TCM) therapies, and attentively listened to my concerns."*

[ ] 1. Strongly disagree

[ ] 2. Disagree

[ ] 3. Neutral

[ ] 4. Agree

[ ] 5. Strongly agree

**E2. TCM Treatment Acceptability**

*Statement: "I found the TCM non-pharmacological therapies and interventions provided during my hospitalization to be highly acceptable, comfortable, and beneficial to my recovery."*

[ ] 1. Strongly disagree

[ ] 2. Disagree

[ ] 3. Neutral

[ ] 4. Agree

[ ] 5. Strongly agree

**E3. Overall Satisfaction**

*Statement: "Overall, I am highly satisfied with the medical care, service quality, and treatment outcomes of this hospitalization."*

[ ] 1. Strongly disagree

[ ] 2. Disagree

[ ] 3. Neutral

[ ] 4. Agree

[ ] 5. Strongly agree

**E4. Future Willingness**

*If you were to experience the same or a similar health condition in the future, would you be willing to choose this specific TCM treatment pathway and payment model again?*

[ ] 1. Yes, definitely

[ ] 2. Yes, probably

[ ] 3. Unsure

[ ] 4. Probably not

[ ] 5. Definitely not

*End of Questionnaire. Thank you for your participation.*
