# Supplementary material for: Aligning insurance payment with public value: outcome-based payment for traditional Chinese medicine advantage diseases under China’s DRG/DIP reform
Source: Front Public Health. 2026 Oct 21;14:1902781. doi: 10.3389/fpubh.2026.1902781 (PMC13730889; doi:10.3389/fpubh.2026.1902781)
Supplement: Supplementary file 2 [file Table_2.DOCX]

**Supplementary Appendix S2**

**Semi-Structured Interview Guide for Key Stakeholders**

**Interview Context and Instructions for Interviewers:**

This semi-structured interview framework is designed to explore the implementation mechanisms, perceived impacts, and practical barriers of the outcome-based payment model for TCM-advantage diseases under the DRG/DIP reform. The interviews aim to contextualize the quantitative findings related to the Patient-Centered Public Value Index (PCPVI).

Given the distinct roles of the 10 recruited stakeholders, the guide is divided into four modules: (1) Patients, (2) TCM Clinicians, (3) Hospital Insurance Management Staff, and (4) Medical Insurance Administrative Personnel. Interviewers should use these questions as a foundational guide, utilizing follow-up probes (e.g., "Could you provide a specific example?", "Why do you think that is the case?") to explore emerging themes until theoretical saturation is reached.

**Module 1: Patients (n=3)**

*Focus: Perceived cost burden, therapeutic value of TCM, and overall care experience.*

1. **Overall Experience & Therapeutic Value:**
   - Could you describe your overall experience with the treatments you received during this hospitalization?
   - How do you perceive the effectiveness and comfort of the Traditional Chinese Medicine (TCM) therapies provided for your condition?
2. **Financial Burden & Insurance Perception:**
   - What are your thoughts on the out-of-pocket costs for this hospitalization? Did the medical insurance reimbursement meet your expectations in terms of reducing your financial burden?
3. **Communication & Acceptability:**
   - Did the medical team clearly explain why specific TCM treatments were chosen for you?
   - Based on this experience, how willing would you be to undergo a similar TCM-focused treatment pathway in the future?

**Module 2: TCM Clinicians (n=3)**

*Focus: Influence of payment reform on clinical behavior, therapeutic value recognition, and documentation workload.*

1. **Clinical Behavior & Pathway Standardization:**
   - How has the introduction of the outcome-based payment pathway for TCM-advantage diseases affected your daily clinical decision-making and your adherence to standardized clinical pathways?
2. **Value Recognition:**
   - Compared to conventional DRG/DIP payment, do you feel this new payment model more accurately captures and rewards the unique therapeutic value and clinical efforts involved in TCM treatments (e.g., non-pharmacological therapies, syndrome differentiation)?
3. **Implementation Barriers & Workload:**
   - What are the primary practical challenges you face when managing patients under this outcome-based model?
   - Specifically, how has the reform impacted your workload regarding medical record documentation, TCM syndrome coding, and quality metric reporting?

**Module 3: Hospital Insurance Management Staff (n=2)**

*Focus: Cost-control pressure, pathway management, and administrative implementation.*

1. **Financial Optimization & Cost Control:**
   - From a hospital management perspective, how does the outcome-based payment model alter internal cost-control pressures compared to standard DRG/DIP settlements?
   - Have you observed any changes in the hospital's financial risk or reimbursement efficiency for these specific disease cohorts?
2. **Service Structure & Quality Management:**
   - How has the policy influenced the proportion of TCM treatment costs and the provision of appropriate TCM technologies within your institution?
3. **Administrative & Systemic Challenges:**
   - What are the main administrative hurdles in implementing this payment pathway?
   - Are there specific difficulties in aligning hospital information systems (HIS) with medical insurance settlement requirements for outcome evaluation?

**Module 4: Medical Insurance Administrative Personnel (n=2)**

*Focus: Fund sustainability, dynamic adjustment of standards, and public value realization.*

1. **Fund Sustainability & Incentive Alignment:**
   - How do you evaluate the current balance between incentivizing high-quality TCM care and maintaining the short- and long-term sustainability of the medical insurance fund?
2. **Dynamic Adjustment Mechanisms:**
   - The effectiveness of outcome-based payment relies on accurate baseline pricing and outcome targets. What mechanisms are currently in place—or need to be developed—for the dynamic adjustment of these payment standards as clinical practices evolve?
3. **Barriers to Public Value Realization:**
   - Based on the implementation data and feedback from hospitals, what do you view as the greatest systemic barriers to fully realizing patient-centered public value (e.g., equity, efficiency, financial protection) under this reform?
   - How might future policy iterations address the risks of institutional cream-skimming or pathway compression?
